# A Plan Suggested

**Published:** 1890-09

**Authors:** 


### A PLAN SUGGESTED.

It would appear as a self-evident proposition that, by every consideration of right, equity and justice, the privilege of practicing medicine should be allowed those only, who are qualified for its intelligent exercise; but, in light of the past failures on the part of the State Medical Association to secure the enactment of a law to that effect, it seems difficult to make the legislators believe it. The trouble has been, as the JOURNAL has before insisted, that these efforts have been spasmodic, and did not represent the unanimous sentiment of the profession, as, witness the "protests" which poured in from members who had not been consulted on the subject, and who therefore, did not understand, and were not in sympathy with, the movement. Many of those who protested are now members of the State Association, and will gladly co-operate in a renewed attempt, if the opportunity be given them.

As the JOURNAL has repeatedly said, not until the profession speaks as one man will any such petition receive at the hands of the legislature a respectful consideration.

Every other right, the right of persons, of property, the pursuit of happiness; every other interest is hedged around with safeguards as strong as words will make them; while the lives of the people are at the mercy (?) and depend, often, upon the word, of some ignorant pretender to the medical science.

The question then arises—how shall the sentiment of the profession be obtained? How can it be made to "speak as one man?" How can the legislature be convinced that the entire educated element of the medical profession of Texas believe that such a law is imperatively demanded in the interest of the public health and safety? And that it is for the good of the people that it is asked—and that they need it and want it?

We submit, it is folly to talk of "suppressing quackery"; too much of it exists, in and out of the profession, too many diploma-ed gents and licensed fellows—"legal practitioners," are at work, and a retro-active law,—one to reach and stop them, is not to be thought of. Surely quackrey—as it exists in its protean form, can never be suppressed by the monthly discharge of personal abuse and invective through the columns of any alleged "journal," however regular, persistent and vulgar. A sustained effort on the part of the medical profession to convince the legislature that a danger—greater than that of epidemic invasion—threatens the public health in the indiscriminate practice of medicine, might elicit a law to require in future—an examination of all who essay to engage in practice,—but such a law even, would not "suppress quackery."

As the JOURNAL has before suggested—a feasible plan by which the sentiment of the profession can be gotten at, would be to have the committee on legislation draw up a petition to the legislature setting forth briefly but forcibly—the situation, and the necessity of reform; and have every county, town, and district medical society in the State to endorse and sign it; then let a committee from each society procure the signatures of every reputable physican—not members—within their several sections—or districts; let these petitions be handed the several representatives—in House and Senate and be simultaneously presented. The legislature will meet in January, and no time should be lost.

But, as we are not of, nor on the committee, we can only suggest this course, and we repectfully do so—to the honorable committee. Should the chairman of the committee, Dr. Cupples—request it, the Secretary of the Association will cause to be printed for the purpose, blanks in such form as may be submitted; there is plenty of money in the treasury to pay for them,—and no better use could be made of it. This is respectfully suggested also.

Indeed some such scheme was mentioned in connection with the very able—but so far—fruitless, report submitted by Chairman Cupples of the legislative committee, at San Antonio, which report, in accordance with instructions, was printed in pamphlet form and 3000 copies were mailed to the leading physician of the State.

The present skeleton of a law could be so amended as to meet the requirements; and perhaps that would be the better plan; at least for the present.

Other things are needed; a law to regulate the sale of poisons, to prevent adulteration of food and drugs, provisions for collecting and preserving vital statistics, etc; but of these more anon.

